# Upper Airway Assessment in Cone-Beam Computed Tomography for Screening of Obstructive Sleep Apnea Syndrome: Development of an Evaluation Protocol in Dentistry

**DOI:** 10.2196/41049

**Published:** 2023-05-05

**Authors:** Catarina Fonseca, Francisca Cavadas, Patrícia Fonseca

**Affiliations:** 1 Faculty of Dental Medicine Universidade Católica Portuguesa Viseu Portugal; 2 Center for Interdisciplinary Research in Health Viseu Portugal

**Keywords:** cone-beam computed tomography, three-dimensional image, 3D image, airway obstructions, sleep medicine specialty, dentistry, obstructive sleep apnea, protocol

## Abstract

**Background:**

The upper airways are formed by the nasal cavities, pharynx, and larynx. There are several radiographic methods that allow evaluation of the craniofacial structure. Upper airway analysis in cone-beam computed tomography (CBCT) may be useful in diagnosing some pathologies such as obstructive sleep apnea syndrome (OSAS). OSAS prevalence has increased significantly in recent decades, justified by increased obesity and average life expectancy. It can be associated with cardiovascular, respiratory, and neurovascular diseases, diabetes, and hypertension. In some individuals with OSAS, the upper airway is compromised and narrowed. Nowadays, CBCT is widely used in dentistry by clinicians. Its use for upper airway assessment would be an advantage for screening some abnormalities related to an increased risk of pathologies such as OSAS. CBCT helps to calculate the total volume of the airways and their area in different anatomical planes (sagittal, coronal, and transverse). It also helps identify regions with the highest anteroposterior and laterolateral constriction of the airways. Despite its undoubted advantages, airway assessment is not routinely performed in dentistry. There is no protocol that allows comparisons between studies, which makes it difficult to obtain scientific evidence in this area. Hence, there is an urgent need to standardize the protocol for upper airway measurement to help clinicians identify at-risk patients.

**Objective:**

Our main aim is to develop a standard protocol for upper airway evaluation in CBCT for OSAS screening in dentistry.

**Methods:**

To measure and evaluate the upper airways, data are obtained using Planmeca ProMax 3D (Planmeca). Patient orientation is performed in accordance with the manufacturer's indications at the time of image acquisition. The exposure corresponds to 90 kV, 8 mA, and 13,713 seconds. The software used for upper airway analysis is Romexis (version 5.1.O.R; Planmeca). The images are exhibited in accordance with the field of view of 20.1×17.4 cm, size of 502×502×436 mm, and voxel size of 400 μm.

**Results:**

The protocol described and illustrated here allows for automatic calculation of the total volume of the pharyngeal airspace, its area of greatest narrowing, its location, and the smallest anteroposterior and laterolateral dimensions of the pharynx. These measurements are carried out automatically by the imaging software whose reliability is proven by the existing literature. Thus, we could reduce the possible bias of manual measurement, aiming at data collection.

**Conclusions:**

The use of this protocol by dentists will allow for standardization of the measurements and constitutes a valuable screening tool for OSAS. This protocol may also be suitable for other imaging software. The anatomical points used as reference are most relevant for standardizing studies in this field.

**International Registered Report Identifier (IRRID):**

RR1-10.2196/41049

## Introduction

The upper airways are formed by the nasal cavities, pharynx, and larynx. The pharynx (portion most susceptible to collapse) is divided into 3 parts: nasopharynx, oropharynx, and hypopharynx [1,2]. The nasopharynx begins in the choanae (posterior opening of the nasal cavities) and ends on the hard palate. The oropharynx ranges from the uvula to the epiglottis. The hypopharynx spans the area from the epiglottis to the vocal cords, where the trachea begins [1,2].

There are several radiographic methods that allow the evaluation of craniofacial structure. Profile teleradiography, cone-beam computed tomography (CBCT), axial computed tomography (CT), and magnetic resonance imaging are some examples of imaging modalities that allow for this evaluation. Among these auxiliary diagnostic means and comparing those that are available to dentists, CBCT has demonstrated usefulness in 3D evaluation of the airways. For example, the time and radiation exposure in CBCT are considerably shorter than those in CT [1-3]. Profile teleradiography provides a quick 2D assessment of the upper airways but can only depict the 2D morphology and has prone of numerous superimpositions [4-6].

Furthermore, CBCT images are not magnified and are recorded on a 1:1 scale, which permits accurate and direct dimensional measurements [4-6]. Although a radiographic examination, CBCT has a far more favorable risk-to-benefit ratio. It helps determine the total volume of the airways and their area in the different anatomical planes (sagittal, coronal, and transverse) and identify the regions with the highest anteroposterior and laterolateral constriction of the airways [1].

Upper airway analysis in CBCT is useful to help diagnose some pathologies such as the obstructive sleep apnea syndrome (OSAS).

The OSAS is defined by repeated episodes, greater than 5 per hour, of partial or total obstruction of the upper airways during sleep, leading to airway obstruction (apnea) or reduced airflow (hypopnea). An apnea event, by definition, should last at least 10 seconds and is usually associated with sleep or microarousal fragmentation. Hypopnea can be defined as a reduction in ventilation (at least 50%) with an oxygen desaturation of ≥4% [4-11].

This pathology has a relevant negative impact on patients' daily lives and its prevalence has increased significantly in recent decades worldwide, justified by increased obesity and average life expectancy. Benjafield et al [12] estimated that around the world, approximately 1 billion adults aged 30-69 years experience OSAS, of whom more than 45% (425 million) of adults present moderate to severe OSAS requiring treatment [8]. There is a wide geographical variation in the prevalence of OSAS, with some countries having a prevalence above 50%. OSAS is highly underrecognized, and it is estimated that 82% of men and 93% of women with OSAS in the United States are not diagnosed [12-14]. The clinical presentation of OSAS includes fatigue, daytime sleepiness, and snoring. If not treated, it can be associated with cardiovascular (coronary artery disease, arrhythmias, hypertension, congestive heart failure, and stroke), respiratory (exacerbation of asthma, respiratory dysfunction in chronic obstructive pulmonary disease, pulmonary embolism, and pulmonary hypertension), and neurological diseases (frustration, distress, neurocognitive dysfunctions, and attention deficit) and diabetes [4-11].

In OSAS, despite respiratory effort, there is a repetitive total or partial collapse of the upper airways. In normal situations, the genioglossal muscle contracts at each inspiration in order to avoid the posterior movement of the tongue aided by the lift and tensor muscles of the palate, geniohyoid, and stylopharyngeus [4-11]. Together, these muscles ensure continuous air passage through the airways. However, in some individuals, the upper airways are compromised and narrowed as a result of the deposition of adipose tissue in the pharyngeal muscles and parapharyngeal pathways or due to anomalies in the craniofacial structure [4-11].

One of the regions relevant to the pathophysiology of OSAS is the oropharynx. Steffy et al [1] related the constriction value of the oropharynx with the risk of developing OSAS. An area of <52 mm^2^ is considered as having a high risk of OSAS; 52-100 mm^2^, intermediate risk; and >100 mm^2^, low risk [1,10]. Obstruction may arise at different levels of the oropharynx, being more frequent in the retropalatal, retroglossal, and epiglottis areas [10,11].

Due to the severity of this syndrome, multidisciplinary intervention is essential. The role of the dentist in OSAS is essentially 2-fold: contributing to early diagnosis and therapy. This clinician has the possibility to make a direct observation of the palate’s soft and oropharyngeal tissues (eg, tonsil region and tongue), can identify breathing difficulties and breathing type (nasal or oral), and has easy access to the cervical region, which helps assess its volume or even measure the cervical perimeter [12]. Furthermore, according to the American Academy of Dental Sleep Medicine, the dentist plays a key role in screening and can help to reduce the number of undiagnosed or untreated patients with OSAS [13].

Currently, CBCT is used in dentistry. Its additional use for upper airway assessment would be an added value for the screening of some abnormalities related to an increased risk of OSAS. Studies on airway measurement methods have revealed that there is no objective and consensual methodology [14,15], which makes it difficult to obtain scientific evidence in this area. There is an urgent need to standardize the protocol and to develop a simple workflow for upper airway measurement, which is the aim of this study.

## Methods

### Ethical Considerations

This study was integrated in the project number 27 of the Faculty of Dental Medicine, Catholic University of Portugal, and approved by the Ethics Committee for Health (272022; January 21, 2022). The authors ensured conditions of anonymity and confidentiality that are required for such studies in Portugal.

### Data Collection

**Figure 1:**
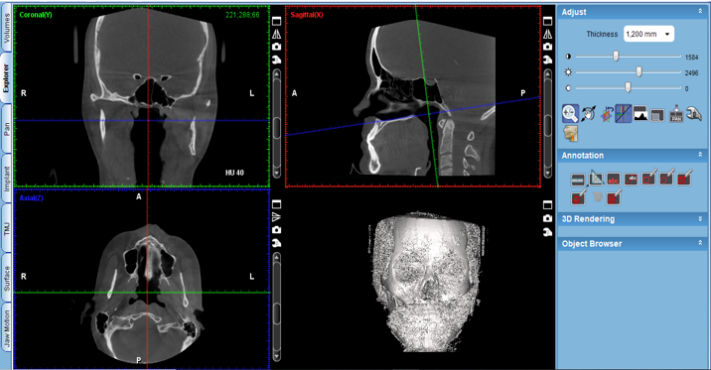
Correct orientation of the planes according to the inclination of the airways.

To measure and evaluate the upper airways, data were obtained using Planmeca ProMax 3D (Planmeca) and in accordance with the manufacturer's indications regarding patient orientation at the time of image acquisition: with the patient standing and with his/her head oriented along 3 vertical references—the facial midline (glabella, subnasal, and mentum), endocant of the eye, and the parietal bone (Figure 1). The patient’s head should also be oriented along a horizontal reference—next to the chin. The exposure corresponds to 90 kV, 8 mA, and 13,713 seconds. Others imaging devices can be used to obtain the CBCT image.

Data collected through CBCT is intended to be used to develop a standard method or protocol for upper airway evaluation.

To develop this protocol, we consulted the radiographic database of the Dental Clinic of the Faculty of Dental Medicine, Catholic University of Portugal, which has a Planmeca ProMax 3D (Planmeca) device. We searched for patients who had complete CBCT (maxilla and mandible) images to perform the analysis.

### Data Analysis

The software used for upper airway analysis is Romexis (version 5.1.O.R; Planmeca). The images are exhibited in accordance with the field of view of 20.1×17.4 cm, size of 502×502×436 mm, and voxel size of 400 μm.

The measurements performed for all the CBCT images were as follows: total volume of the pharyngeal airspace, its area and its location, and the smaller dimensions of the anteroposterior and lateral pharynx (these measurements are illustrated in the *Results* section). 

**Figure 2:**
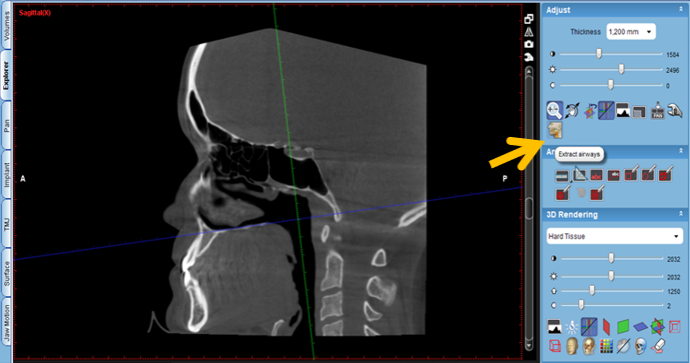
Selection of the tool "Extract Airways" and marking of the points following the curvature of the airways.

**Figure 3:**
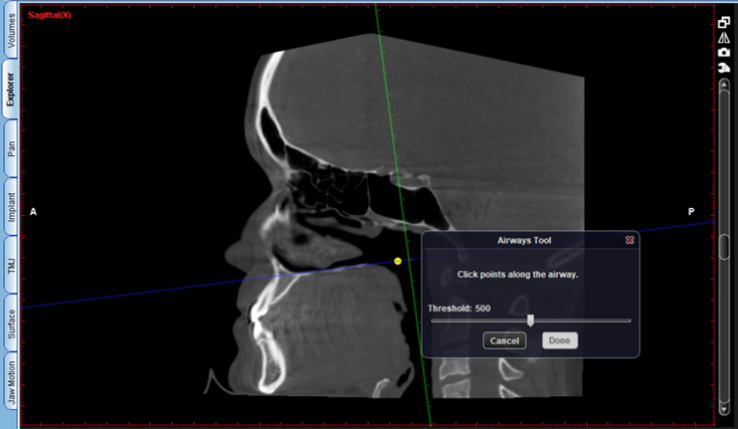
Keeping the threshold at 500 and marking the first point.

**Figure 4:**
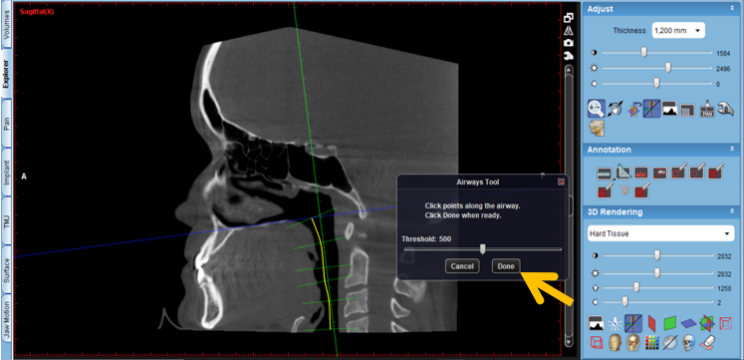
Delimitation of the airway following its curvature.

**Figure 5:**
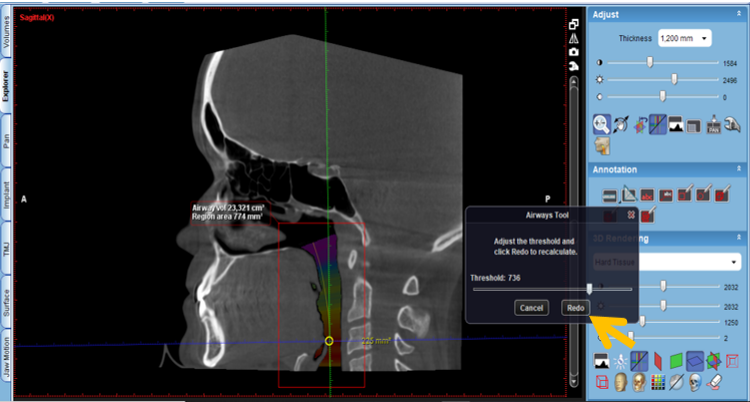
New selection of the option "Extract airways," and in the box that appears with the name "Airways Tool" the variable that defines the image limit is adjusted (standardized value for all patients=736).

**Figure 6:**
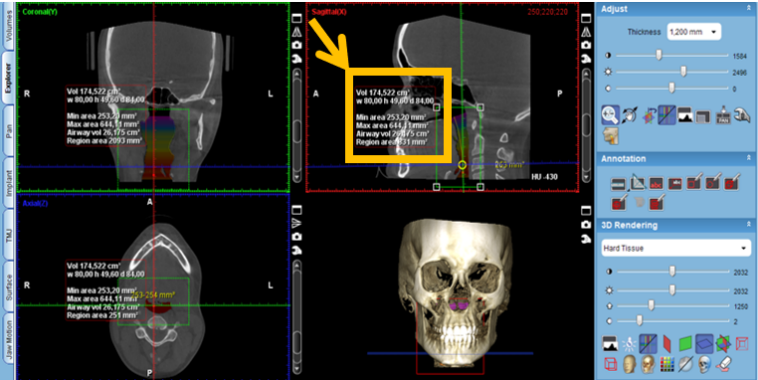
Calculation of the value of the total volume and the value of the minimum airway area ("min area").

**Figure 7:**
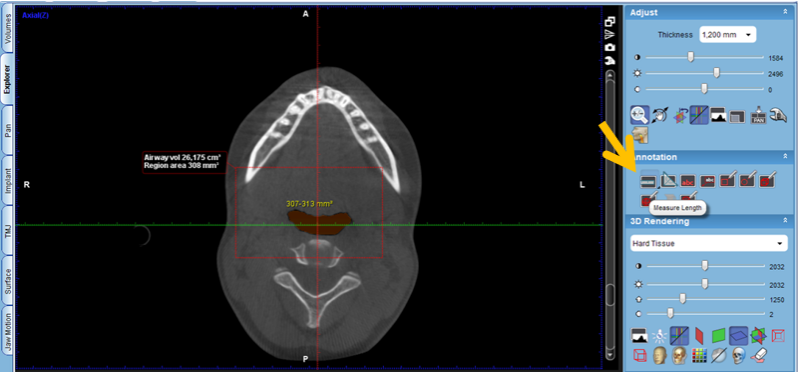
Anteroposterior dimension measurement.

The protocol is as follows:

1. Activate the “Extract airways” tool in the box on the right with the title “Adjust” (Figure 2).
2. Then, enlarge the sagittal cut window in the box that appears on the “Airways Tool” screen to keep the threshold at 500 and mark the first point at the level of the posterior nasal spine (Figure 3).
3. Then, continue delimiting the airway following its curvature and mark a last point at the level of the middle of the fourth cervical vertebra (Figure 4).
4. Select, again, the option “Extract airways,” and in the box that appears with the name “Airways Tool” adjust the variable that defines the airway limits (a standard adjustment value should be used for all patients so that it is possible to establish adjustments; in this case, the standardized value was 736). Then, the value of the total volume (“airway volume”) appears (Figure 5).
5. Place the cursor over the box where the total volume value appears. In the same box will appear the value of the minimum airway area (“min area”) (Figure 6).
6. Enlarge the window containing the axial plane and, from the narrowest area of the airway, select in the box on the right with the heading “Annotation” the tool “Measure Length” and measure the anteroposterior and laterolateral dimensions (Figure 7).
7. Record all obtained values.

To ensure the reproducibility of the protocol, all researchers performed the measurements, and patients were categorized into those at risk for OSAS (highest anteroposterior and laterolateral constriction of the airways of <52 mm^2^ or 52-100 mm^2^) or those at no risk (>100 mm^2^).

## Results

To establish this protocol, CBCT images of 30 patients were used (56% for males and 44% for females), and, in cases where an area of <52 mm^2^ or 52-100 mm^2^ was detected, the patient was contacted and referred to the hospital for diagnostic confirmation tests.

No differences were found between measurements obtained by the different researchers following the protocol described.

The protocol that the authors suggested for evaluation of the upper airways in CBCT allows for automatic calculation of the total volume of the pharyngeal airspace, its area of greatest narrowing, its location, and the smallest anteroposterior and laterolateral dimensions of the pharynx [15-18]. The measurement is performed automatically by the imaging software whose reliability is proven by the existing literature [16]. Thus, we reduce the possible bias of manual measurement, aiming at data collection.

## Discussion

### Principal Findings

The literature revealed no consensual and objective methodology for upper airway measurement. The lack of standardization in the definition of anatomical limits makes it impossible to subsequently make comparisons between different studies [19,20]. Recently, with the evolution of software, it is possible to perform upper airway measurement in a fast automatic analysis in an accurate, reproductible, and practical way. Its simplicity and speed allow this analysis to be adopted by all professionals. It is important to note that simplicity is not achieved in the detriment of any decrease in viability and reliability in the measurement of airways [16].

The CBCT helps measure the total volume of the pharyngeal airspace, its area of greatest narrowing in 3 different anatomical planes (sagittal, coronal, and transversal), its location, and the smallest anteroposterior and laterolateral dimensions of the pharynx [14-16]. Steffy et al [1] related the constriction value of the oropharynx with the risk of developing OSAS. An area of <52 mm^2^ is considered as having a high risk of OSAS; 52-100 mm^2^, intermediate risk; and >100 mm^2^, low risk [1]. Compared to the other radiographic methods described in the literature, usually available to the dentist, such as profile teleradiography and CT, the CBCT, as previously mentioned, presents more advantages. It is possible to acquire the same or better images with less radiation and less time of exposure. The assessment protocol for CBCT described herein constitutes a valuable screening tool for OSAS, which allows clinicians to refer the patient to the hospital for diagnosis confirmation. To establish this protocol, CBCT images of 30 patients were used, and in cases where an area of <52 mm^2^ or 52-100 mm^2^ was detected, the patient was contacted and referred to the hospital for diagnostic confirmation tests.

### Limitations and Strengths

The protocol’s main limitations are related to the position at which this examination is performed. The patient undergoes the examination in a standing position and not in a supine position, which may introduce false negatives here or cause a suspected hypothesis to be discarded. In addition, the step responsible for adjusting the threshold is characteristic of each examination. This threshold adapts the difference between the shades of gray in each voxel of the airway and the rest of the area. However, in general, Zimmerman et al [19] verified, in a systematic review, that most studies measuring the airways have high intraexaminer reliability. Although in this study, no CBCT image was purposely acquired, currently, it is known that it is a comprehensive examination that allows numerous interpretations and simulations from a very low radiation dose. Nevertheless, the ALARA (ie, *As Low As Reasonably Achievable*) principle should be respected, reserving the execution of these examinations for cases in which they are strictly necessary [19,20].

However, despite these limitations and due to the automatic measurements performed by the system, this protocol in CBCT constitutes a good patient screening tool that will help standardize the measurements and ensure that they are performed in a reproductible, accurate, and simple way while the comfort of and benefit for the patients increases.

### Conclusions

This protocol provides a new standardized tool to identify patients at the risk of OSAS by using CBCT and contributes to the detection of upper airway–related pathologies, such as OSAS, in patients visiting the dental clinics, using these devices and software. This protocol will have a significant impact in reducing the subdiagnosis of this pathology, contributing to its treatment and subsequently the prevention of serious comorbidities associated with OSAS. Our protocol may also be suitable for other imaging software. The anatomical points used for reference are most relevant for standardizing studies in this field and for software evaluation to reduce bias.

## Abbreviations

CBCT: cone-beam computed tomography
CT: computed tomography
OSAS: obstructive sleep apnea syndrome
